# Orally administered branaplam does not impact neurogenesis in juvenile mice, rats, and dogs

**DOI:** 10.1242/bio.058551

**Published:** 2021-10-28

**Authors:** Diethilde Theil, Reginald Valdez, Katy Darribat, Arno Doelemeyer, Rajeev Sivasankaran, Andreas Hartmann

**Affiliations:** 1 Translation medicine/preclinical safety, Novartis Institutes for Biomedical Research, Basel 4058, Switzerland; 2 Neuroscience/rare diseases, Novartis Institutes for Biomedical Research, Cambridge, MA 02139, USA

**Keywords:** Imaging, Immunohistochemistry, Mouse, Rat, Dog, Neurogenesis

## Abstract

Branaplam is a therapeutic agent currently in clinical development for the treatment of infants with type 1 spinal muscular atrophy (SMA). Since preclinical studies showed that branaplam had cell-cycle arrest effects, we sought to determine whether branaplam may affect postnatal cerebellar development and brain neurogenesis. Here, we describe a novel approach for developmental neurotoxicity testing (DNT) of a central nervous system (CNS) active drug. The effects of orally administered branaplam were evaluated in the SMA neonatal mouse model (*SMN*Δ*7*), and in juvenile Wistar Hannover rats and Beagle dogs. Histopathological examination and complementary immunohistochemical studies focused on areas of neurogenesis in the cerebellum (mice, rats, and dogs), and the subventricular zone of the striatum and dentate gyrus (rats and dogs) using antibodies directed against Ki67, phosphorylated histone H3, cleaved caspase-3, and glial fibrillary acidic protein. Additionally, image-analysis based quantification of calbindin-D28k and Ki67 was performed in rats and dogs. The patterns of cell proliferation and apoptosis, and neural migration and innervation in the cerebellum and other brain regions of active adult neurogenesis did not differ between branaplam- and control-treated animals. Quantitative image analysis did not reveal any changes in calbindin-D28k and Ki67 expression in rats and dogs. The data show that orally administered branaplam has no impact on neurogenesis in juvenile animals. Application of selected immunohistochemical stainings in combination with quantitative image analysis on a few critical areas of postnatal CNS development offer a reliable approach to assess DNT of CNS-active drug candidates in juvenile animal toxicity studies.

## Abstract (precis)

**Summary:** Branaplam exhibits effects on the cell cycle but does not impair neuronal proliferation in juvenile or young adult animals, as demonstrated by immunohistochemistry and automated digital imaging.

## INTRODUCTION

Spinal muscular atrophy (SMA) is an autosomal recessive neurodegenerative disease that is characterized by deficiency in the survival motor neuron (SMN) protein, often the result of the deletion of gene *SMN1*, that leads to progressive muscle weakness (Burghes and Beattie, 2009; Kolb and Kissel, 2015; Lunn and Wang, 2008; Tizzano and Finkel, 2017). SMA is rare, occurring in 1 in 11,000 births (Sugarman et al., 2012), and patients with the more severe form (type 1) die before the age of 2 years if they do not receive treatment (Swoboda et al., 2005). The first treatments for SMA were approved in 2016 [nusinersen (Spinraza)] (Spinraza Prescribing Information, 2021) and 2019 [onasemnogene abeparvovec (Zolgensma)] (Zolgensma Prescribing Information, 2021), and additional treatments are currently in clinical trials (Ramdas and Servais, 2020). Most recently, Risdiplam (Evrysdi), an orally administered RNA splice-modifying small molecule developed by Roche, was recently approved by the FDA for all types of SMA (FDA News Release, 2020). Currently, branaplam, another oral small molecule, is being evaluated in an open-label, phase 1/2 trial in infants with type 1 SMA (NCT02268552), and has been shown to have good efficacy, safety, and tolerability (Charnas et al., 2017; Jevtic et al., 2019).

Branaplam is an mRNA splicing corrector. Specifically, branaplam modulates *SMN2* splicing and increases generation of full-length *SMN2* mRNA and functional SMN protein. This *SMN2* splicing modulation results because of the sequence-selective increased binding affinity of U1 small nuclear ribonucleic protein (snRNP) to the 5′ splice site of *SMN2* in the presence of branaplam (Cheung et al., 2018; Palacino et al., 2015). Preclinical studies show that branaplam is distributed to the brain in mice (Palacino et al., 2015) and is expected to be distributed similarly in humans. Branaplam was also shown to have adverse effects, such as cell-cycle inhibition and aneugenic effects both *in vitro* and *in vivo*.

The evaluation of potential developmental neurotoxicity (DNT) is an essential part of drug safety assessment, especially for compounds that specifically target the central nervous system (CNS) intended for use in pediatric patients. Current guidelines for DNT studies refer to drug exposure during gestation and early postnatal development. This includes functional and behavioral endpoints, as well as gross and qualitative histological assessments allowing for detection of deviations of normal anatomy such as hypoplasia or heterotopias (Organization for Economic Co-operation and Development, 2007, 2011, 2015; United States Environmental Protection Agency, 1998). The DNT guideline OECD TG 426 applies to exposure from gestation day 6 to postnatal day 21, assessing exposed animals through maternal milk from birth until weaning. Similarly, the ICH S5 (R2) (2005) safety guidance applies to exposure from implantation to weaning. The current test guidelines do not specifically address juvenile drug exposures and effects; however, subtle toxic developmental abnormalities may occur in juvenile and adolescent animals that may be missed if not assessed by appropriate means (Garman et al., 2015; Li et al., 2017). It is prudent that tailored DNT testing is performed in juvenile animals according to identified adverse (off-target) effects of compounds. In the case of compounds having effects on the cell cycle, we believe that DNT studies should be extended to include assessment of neurogenesis and neuronal migration in juvenile animals.

In order to assess potential effects of branaplam in juvenile animals at stages when brain development is still ongoing, we developed a novel approach for DNT testing that targets selected areas of the CNS known to be important active sites of postnatal development, including neurogenesis and neuronal migration. The selected brain regions for DNT testing in the present study included the cerebellar cortex and regions where neurogenesis continues throughout adult life, such as the subgranular zone (SGZ) of the dentate gyrus of the hippocampus and the subventricular zone (SVZ) of the striatum (Fuentealba et al., 2012; Ming and Song, 2011; Shigemoto-Mogami et al., 2014). In the present study, we assessed the effects of orally administered branaplam on postnatal cerebellar neurogenesis and neuronal migration in juvenile rats and dogs. Since these animal species do not carry the *SMN2* gene, cerebellar neurogenesis was additionally assessed in neonatal *SMN*Δ*7* mice expressing human *SMN2* (Le et al., 2005).

## RESULTS

Neuropathological assessment did not reveal changes indicating abnormal brain development in mice rats, or dogs.

### Immunohistochemical staining results: proliferation markers in the cerebellum

#### SMNΔ7 Mice

**Fig. 1.**
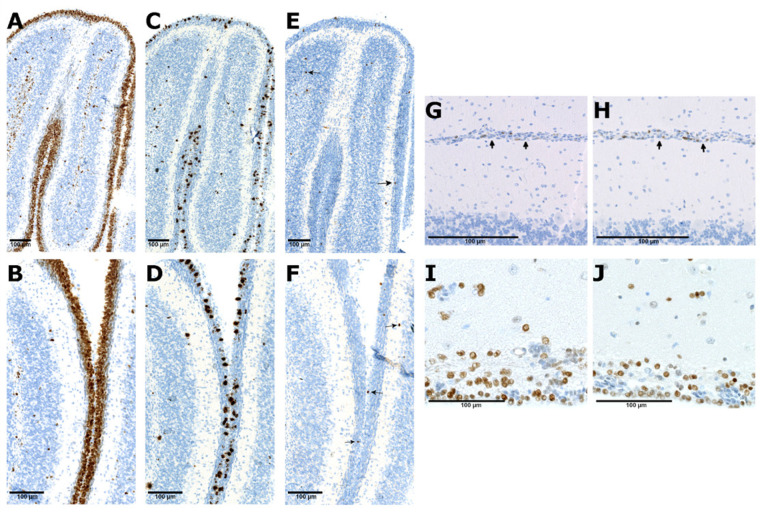
**Ki67, phosphorylated histone 3, and cleaved caspase-3 immunohistochemistry stainings in the cerebellum of the SMA mouse model and juvenile dogs treated with vehicle and branaplam.** Ki67 staining in the external granular layer (EGL, brown stained layer) in the cerebellum of the SMA mouse treated with vehicle (A) and branaplam (B); phosphorylated histone 3 staining (dark brown punctate staining) in the vehicle (C) and branaplam (D) treated mice; cleaved caspase-3 staining (arrows point at single positive cells) in the vehicle (E) and branaplam (F) treated mice; Single Ki67 positive cells in the residual EGL (arrows) in the cerebellum of vehicle (G) and branaplam (H) treated dogs. Numerous cleaved caspase-3 positive cells (brown stained nuclei) in the subventricular zone (SVZ) of vehicle (I) and branaplam (J) treated dogs. Examples shown of three brains assessed per dose group (mice) or ten per dose group (dogs).

In study 1, numerous Ki67 and phosphorylated histone 3 (PHH3) positive cells were found in vehicle- and branaplam-treated animals on postnatal day (PND) 9 (Fig. 1). Ki67 immunoreactivity was observed throughout the entire external granular layer (EGL) band and some cells in the internal granular layer (IGL). Based on microscopic qualitative assessment, no change in the EGL size or distribution of Ki67 and PHH3 in the IGL was noticed in the vehicle-treated mice (Fig. 1A,C) and branaplam-treated mice (Fig. 1B,D). The staining pattern suggests normal postnatal cerebellar development and no effect on mitosis.

In study 2, assessment of Ki67 and PHH3-stained tissue sections did show rare positive cells in branaplam-treated mice at PND 35 and 49. A few Ki67 and PHH3 positive cells were found in the residual EGL of vehicle-treated mice that survived only until PND 14, 15, and 16. The staining pattern suggests normal regression of the EGL in branaplam- and vehicle- treated animals with no changes in proliferation or mitosis (Fig. S1).

#### Wistar Hannover rats

No PHH3 positive proliferating cells were found in the cerebellum at the site of former EGL or IGL at PND 36. Normal regression of the EGL was observed in all branaplam treated rats at all dose levels. The staining pattern was similar with the one observed in the vehicle-treated animals and is compatible with normal proliferation and postnatal cerebellar development (data not shown).

#### Beagle dogs

Proliferating Ki67 positive cells were found in the residual EGL, corresponding to the normal stage of cerebellar development at PND 57. A few PHH3 positive cells were found in the residual EGL. No changes were observed in the proportion of Ki67 and PHH3 positive cells. No changes were observed in the Ki67 and PHH3 the staining pattern in the cerebellum of vehicle-treated (Fig. 1G) and branaplam-treated animals (Fig. 1H). The staining pattern suggests normal proliferation and postnatal cerebellar development.

### Immunohistochemical staining results: proliferation markers in the subventricular zone (SVZ) and the subgranular zone (SGZ)

#### Beagle dogs

**Fig. 2.**
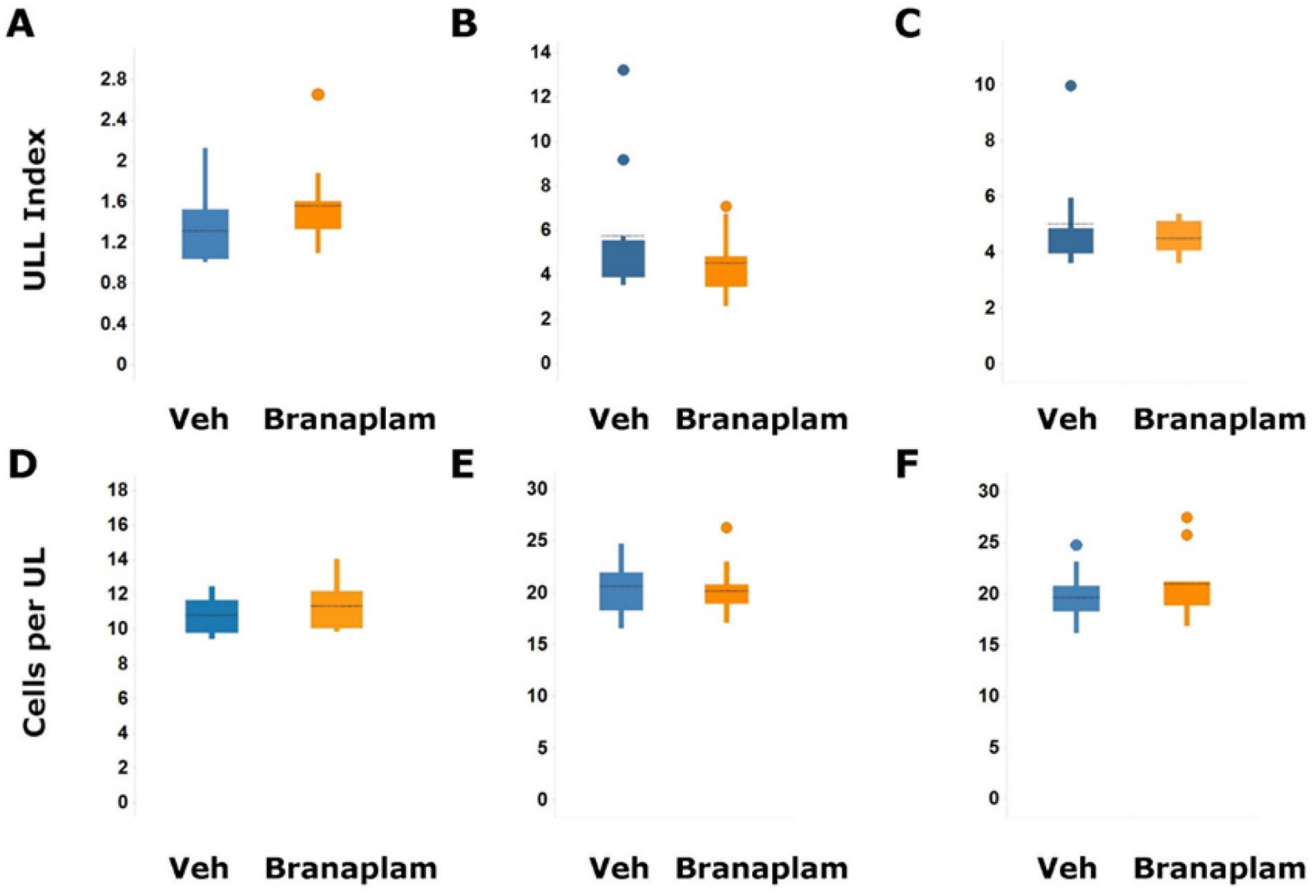
**Image analysis based quantification of Ki67 and Calbindin D-28k positive staining in the striatum (SVZ) and cerebellum.** Quantification of Ki67 in the SVZ of the striatum in the dogs treated with vehicle (*n*=8) and branaplam at 2 mg/kg/day (*n*=10) for 13 weeks (A) and rats treated with vehicle (*n*=10), branaplam (*n*=9) at 2 mg/kg/day for 13 weeks (B) and vehicle (*n*=9) at 2.5 mg/kg/day for 26 weeks (*n*=10) (C). The Ki67-positive area divided by the length of the region of interest was used as a unit length labeling (ULL) index. Quantitative assessment of the length of the molecular layer and estimation of the number of calbindin D-28K-positive Purkinje cells in the cerebellum of dogs treated with vehicle (*n*=10) and branaplam at 2 mg/kg/day (*n*=10) for 13 weeks (D) and rats treated with vehicle (*n*=10) and branaplam at 2 mg/kg/day (*n*=10) for 13 weeks (E) and vehicle (*n*=10) at 2.5 mg/kg/day for 26 weeks (*n*=9) (F). The number of calbindin D-28K-positive cells divided by the length was also used as the readout (cells per unit length); Veh, vehicle. Median of values shown. Significance assessed by Mann–Whitney test.

Proliferating single or clustered Ki67 positive cells and few isolated PHH3 cells were found in the SVZ of the lateral ventricle and in the RMS if it was present on the sections. The staining pattern for both markers was similar in branaplam-treated dogs. No difference in the proportion of Ki67 and PHH3 was observed in vehicle-treated compared with branaplam-treated dogs (Fig. S2). Image-analysis-based quantification in the SVZ did no show a statistically significant difference in the unit length labeling index for Ki67 positive areas (Fig. 2A). Only a few isolated Ki67 and PHH3 positive cells were present in the SGZ of the dentate gyrus with a similar pattern of distribution in dogs treated with vehicle or 2 mg/kg/day branaplam for 13 weeks.

#### Wistar Hannover rats

Abundant proliferating Ki67 positive cells and only few PHH3 positive cells were found in the SVZ and rostral migratory stream (RMS), and olfactory tubercle when present in sections. No difference in proliferating cells was found in the SVZ on PND 36 in rats treated with vehicle or branaplam up to 10 mg/kg (Fig. S3A).

Image analysis based quantification of Ki67 in the SVZ did not reveal any statistically significant difference in the unit length labeling index for Ki67 positive areas in rats treated with vehicle and branaplam at 2 mg/kg/day for 13 weeks and 2.5 mg/kg/day for 26 weeks (Fig. 2B,C and Fig. S3B). Only a few isolated Ki67 and PHH3 positive cells were found in the SGZ of the dentate gyrus with a similar pattern of distribution in rats treated with vehicle or 2 mg/kg/day branaplam for 13 weeks or 2.5 mg/kg/day branaplam for 26 weeks (Fig. S3C).

### Immunohistochemical staining results: apoptosis, Purkinje cell and glia cell alignment

### Apoptosis

#### SMNΔ7 mice

Cleaved caspase-3 positive cells were occasionally seen in the EGL and more frequent in the IGL. No difference in the distribution of caspase-3 positive cells was noticed in vehicle-treated (Fig. 1E) and branaplam-treated mice (Fig. 1F). The staining pattern is compatible with the normal cell death that occurs in the developing cerebellum.

#### Wistar Hannover rats and Beagle dogs

Numerous cleaved caspase-3 positive cells were observed in the SVZ and brain parenchyma of rats and dogs. An example of cleaved caspase-3 positive staining in the SVZ is shown from dogs treated with vehicle (Fig. 1I) and branaplam (Fig. 1J). There was no difference in staining pattern between vehicle and branaplam-treated rats and dogs in all studies (data not shown). The staining pattern is compatible with normal cell death that occurs in the SVZ and brain parenchyma in rats and dogs.

### Purkinje cell and glia alignment

#### Neonatal SMNΔ7 mice

**Fig. 3.**
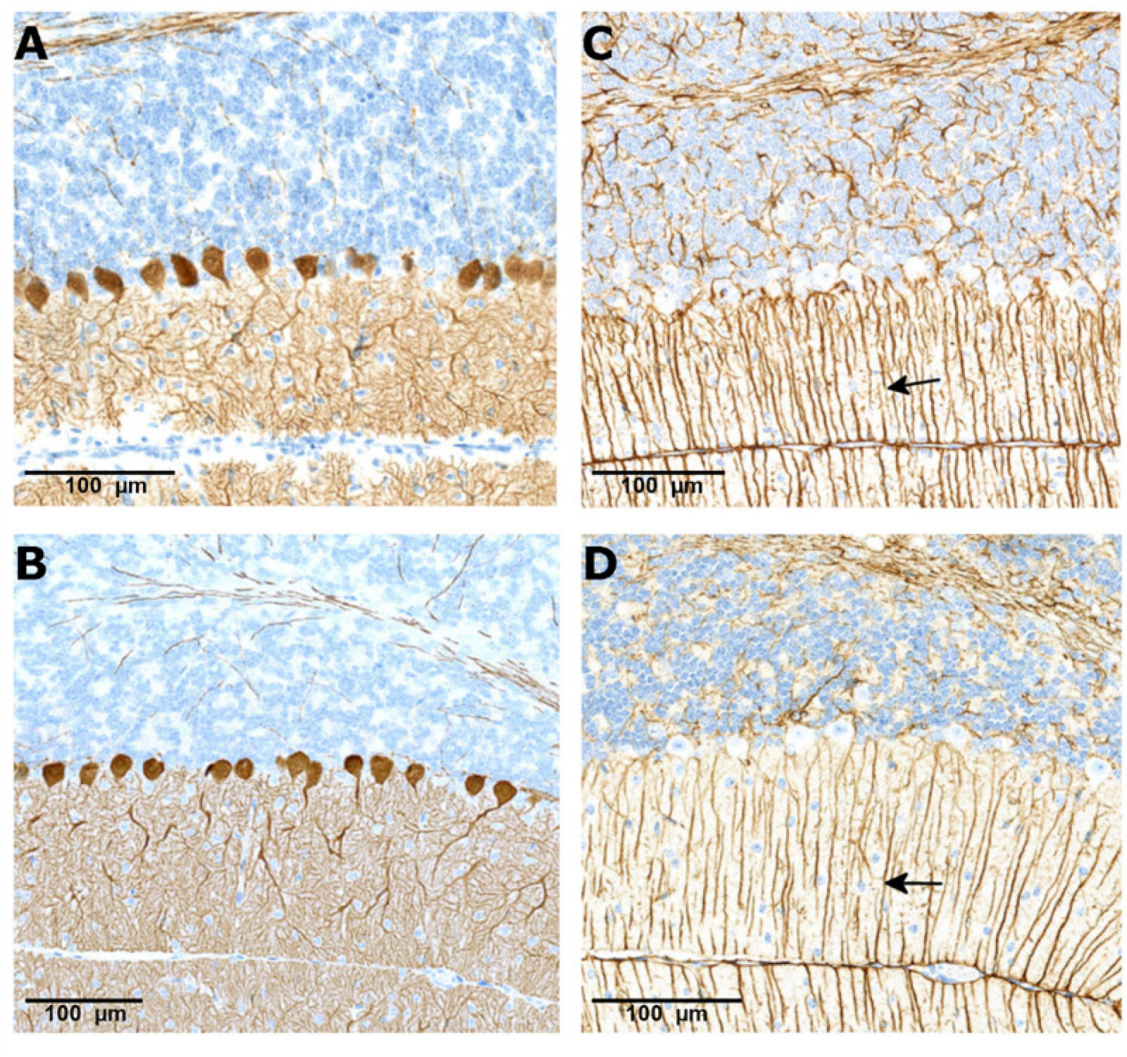
**CalbindinD-28k and GFAP staining in the cerebellum of the SMA mouse model treated with vehicle and branaplam.** CalbindinD-28k staining showing Purkinje cell alignment (brown staining) in a vehicle (A) treated animal at PND 14 and branaplam (B) treated animal at PND 49. Glial fibrillary acidic protein staining showing normal arrangement of Bergman glial fibers (arrows) in the vehicle- (C) and branaplam-treated animal (D). PND, postnatal day.

Expression of calbindin D-28k in the cerebellum of *SMNΔ7* mice was confined to Purkinje cells and their dendritic branches in the molecular layer; treatment with branaplam did not change this normal pattern in both mouse studies. An example of the calbindin D-28k staining is shown in a vehicle-treated mouse PND 14 (Fig. 3A) and branaplam-treated mouse PND 49 (Fig. 3B). Glial fibrillary acidic protein (GFAP) staining showed normal arrangements of Bergman glial fibers in vehicle- and branaplam-treated *SMNΔ7* mice. An example of the GFAP staining is shown in a vehicle-treated mouse PND 14 (Fig. 3C) and branaplam-treated mouse PND 49 (Fig. 3D).

#### Juvenile Wistar Hannover rats

The calbindin D-28k staining in rats indicated normal alignment of Purkinje cells and normal organization of the layers of the cerebellar cortex in all studies on PND 35, PND 98, and PND 189 (Fig. S4A,B). Image analysis was applied on calbindin D-28k stained brain sections from the 13-week and 26-week studies. Assessment of the unit-length labelling index for calbindin D-28k stained Purkinje cells indicated no significant difference between values for rats treated with vehicle or 2 mg/kg/day branaplam for 13 weeks or treated with vehicle or 2.5 mg/kg/day branaplam for 26 weeks, respectively (Fig. 2E,F). Staining for GFAP revealed normal arrangement of the radial glia and no glia scarring. The staining pattern was similar between animals treated with vehicle or branaplam for rats treated with vehicle or 2 mg/kg/day branaplam for 13 weeks (Fig. S4C).

#### Juvenile Beagle dogs

Positive calbindin D-28k staining was observed in the cerebellum in Purkinje cells and in the molecular layer at comparable intensities in the vehicle- and branaplam-treated dogs of all studies. Quantitative assessment of the length of the molecular layer and estimation of the number of Purkinje cells indicated no significant difference between the unit-length labelling indices for these cells in the cerebellum of the vehicle- and branaplam-treated dogs (Fig. 2D). Normal Purkinje cell alignment and molecular layer width were also observed in vehicle- and branaplam-treated dogs from both studies (Fig. S5A) Staining for GFAP revealed normal arrangement, no glia scarring or activation. The staining pattern was similar between animals treated with vehicle or branaplam from both dog studies included in the assessment (Fig. S5B).

### Assessment of genotoxicity potential

#### In vitro

In a mammalian mutagenicity test using human lymphocytes, branaplam induced a concentration-related increase in micronucleus formation. Assessment by fluorescence *in situ* hybridization (FISH) demonstrated that the micronuclei were centromere positive, demonstrating an aneugenic but not clastogenic effect (Table 1).

**Table 1.**
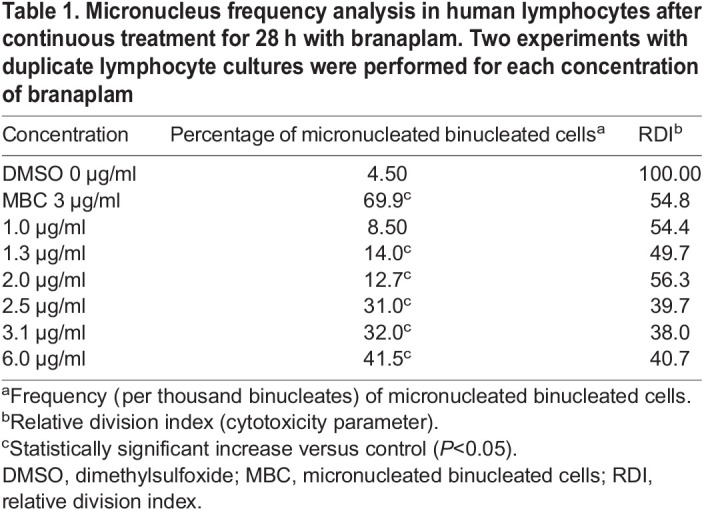
Micronucleus frequency analysis in human lymphocytes after continuous treatment for 28 h with branaplam. Two experiments with duplicate lymphocyte cultures were performed for each concentration of branaplam

#### In vivo

The study in adult male rats showed that branaplam caused an increase in micronucleus frequency at doses of ≥5 mg/kg with a no-observable-effect level (NOEL) of 1 mg/kg. In blood samples collected from a 13-week repeated dose study in juvenile rats, the increased micronucleus frequencies were observed in male rats at a dose of 2.0 mg/kg/day; no such effect was observed in female rats. After a drug-free period of 4 weeks, the micronucleus frequencies in branaplam-treated rats were not different from control rats indicating full reversibility of this change. The NOELs were 0.75 and 2.0 mg/kg in male and female rats, respectively (data not shown).

### Pharmacokinetics

Juvenile rats and dogs administered vehicle had no measurable concentrations of branaplam in plasma, cerebrospinal fluid (CSF) or brain tissue samples. Following repeated oral administration to neonatal *SMNΔ7* mice, juvenile rats, and juvenile dogs, branaplam was quantifiable in all matrices investigated (Table 2). The mean concentrations were calculated in all matrices across both sexes and are presented in Table 2 together with the concentration ratios between brain and plasma or CSF and plasma. Branaplam was distributed into the brain with brain-plasma concentration ratios between 2.8 and 4.3 for the *SMNΔ7* mouse, 2.6 and 3.1 juvenile rat, and 8.9 to 9.9 for the juvenile dog. The CSF-plasma ratios in juvenile dogs ranged between 0.16 and 0.17.

**Table 2.**
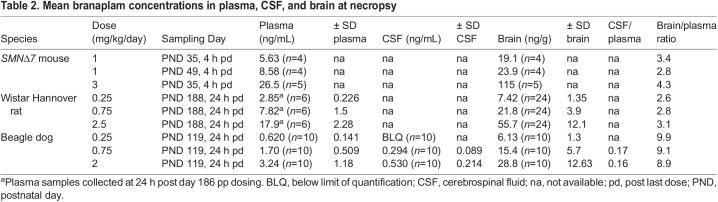
Mean branaplam concentrations in plasma, CSF, and brain at necropsy

## DISCUSSION

Here we presented the results for a novel approach for DNT using the drug candidate branaplam, which showed cell-cycle effects in preclinical safety studies. Specifically, neonatal *SMNΔ7* mice, juvenile Wistar Hannover rats, and juvenile Beagle dogs orally dosed with branaplam showed no signs of aberrant proliferation, apoptosis, or cell-cycle arrest. Similar patterns of cell proliferation (demonstrated by Ki67 and PHH3 staining), and apoptosis (demonstrated by staining for cleaved caspase 3) were observed in vehicle- or branaplam-treated animals. Results obtained with calbindin-D28k immunohistochemistry indicated no morphological abnormalities in Purkinje cell bodies or their dendrites in the molecular layer. Purkinje cell or Bergmann glia misalignment (the latter demonstrated with GFAP staining) was not observed in any species tested, and there was no evidence of glial cell activation or scarring. Distribution of branaplam into the brain was consistent with that previously described for mice and rats after oral administration (Palacino et al., 2015). These results indicate no adverse effects of branaplam on cerebellar architecture and adult neurogenic sites in neonatal mice, juvenile rats, and juvenile dogs detectable by immunohistochemistry staining and image-analysis-based quantification.

Since pediatric patients with SMA are to be treated with branaplam, we wanted to establish a sensitive assessment of potential DNT and brain development in view of the cell-cycle-inhibiting potential of branaplam. We aimed at performing sensitive DNT investigations in the brain of juvenile animals treated with branaplam that were beyond the recommended qualitative histological assessments (Bolon et al., 2018). To achieve this goal, we developed a novel protocol focusing on a few critical areas of postnatal development using immunohistochemistry to assess neurogenesis, neuronal migration and alignment complemented with image analysis-based quantification. Some of these immunohistochemistry stainings were shown to be sensitive in detecting DNT in mice exposed to phenytoin on PND 2 to PND 4, exhibiting abnormal dendritic arborization of Purkinje cells, and persistence of the EGL (Ohmori et al., 1999). Similarly, early postnatal exposure to cisplatin resulted in derangement of glial fibers as demonstrated by light microscopy (Pisu et al., 2005).

In our experiments, branaplam was administered to neonatal and juvenile experimental animals at ages that correspond to the time during which the cerebellum is still developing in humans (Watson et al., 2006). The vast majority of cells in the external germinal zone (EGZ), a temporary population of proliferating cerebellar cells located at the subpial surface of the developing cerebellum, differentiate into granule cells that migrate to form the IGL during the postnatal period (Marzban et al., 2015). In mice, the peak of granule cell production in the EGZ is on PND 7 and proliferation and migration are largely completed by PND 20 (Heng et al., 2011). In rats, EGZ volume peaks at PND 15 and disappears by PND 24 (Heinsen, 1977; Marzban et al., 2015). In dogs, the EGZ is apparent by PND 2 and it persists until the tenth PND week (Phemister and Young, 1968). For comparison, in humans the EGZ disappears between 12 and 18 postnatal months and the cell density in the internal granular layers reaches adult levels by about 2 years of age (Gadsdon and Emery, 1976). Likewise, the SVZ, and SGZ of the hippocampal gyrus dentatus have been recognized as important sites of neurogenesis in the postnatal brain in humans and other mammals, but these anatomical structures are not as well characterized compared to the cerebellum. In humans, neurogenesis in the dentate gyrus of the hippocampus decreases rapidly during the first years of life (Sorrells et al., 2018), whereas in the rat granule cell development occurs between birth and PND 21, and then continues at a lower rate throughout the animal's life (Altman and Bayer, 1990).

These findings are important as clinical studies with the orally administered *SMN* splicing small molecule modulators branaplam and risdiplam show promising efficacy in SMA Type 1 patients; however, modulation of mRNA splicing has safety risks that are believed to be off-target effects (Dunant, 2020; Jevtic et al., 2019; Ratni et al., 2018). Both *SMN* splicing modulators branaplam and risdiplam showed cell-cycle effects, which could be detected with high sensitivity in mammalian cytogenetics assay which are the gold standard in preclinical safety testing. For risdiplam it was postulated that regulation of secondary targets involved in the cell cycle may be responsible for the aneugenic potential (Ratni et al., 2018; Risdiplam Prescribing Information, 2020), whereas the cell-cycle arrest effects of branaplam are probably caused by a stabilizing effect on microtubulin polymerization. Safety findings related to a cell-cycle arrest were observed with both compounds in organs with active proliferation (e.g. gastrointestinal tract and testis) in experimental animals. Additionally, branaplam induced a minimal to mild peripheral axonopathy in dogs in a chronic 52-week juvenile toxicity study and risdiplam caused retina degeneration in a 39-week chronic cynomolgus monkey study (Ratni et al., 2018; Risdiplam Prescribing Information, 2020). Toxicities for these compounds were monitored closely in the clinic, and no peripheral neurotoxicity or retinal toxicity have been reported in patients treated with branaplam or risdiplam (Day et al., 2020; Jevtic et al., 2019; Risdiplam Prescribing Information, 2020).

Our studies demonstrated that orally administered branaplam, under conditions of the studies performed and described here, had no impact on neurogenesis in juvenile mice, rats, or dogs. The immunohistochemistry stainings applied in our studies were robust and reproducible in all species tested, that is, all markers were well established for each species and no stainings were comparable from different IHC runs. Moreover, we also demonstrated that the immunohistochemistry staining was suitable for automated quantitative assessment. Applying these techniques to selected areas critical for postnatal CNS development may offer a reliable and robust approach to assess DNT particularly of CNS active drug candidates for use in pediatric populations.

## MATERIALS AND METHODS

### Animal studies

All animal experiments [neonatal *SMN*Δ*7* mice (a transgenic mouse model of SMA, Le et al., 2005) juvenile Wistar Hannover rats (*Rattus norvegicus*), and juvenile Beagle dogs (*Canis lupus familiaris*)] were reviewed and approved by the respective Institutional Animal Care and Use Committees of either Novartis Institutes for BioMedical Research (Cambridge, MA, USA), PsychoGenics, Inc. (Paramus, NJ, USA) or Charles River Laboratories, Inc. (Montreal, Quebec, Canada); all experiments conformed to the Guide for the Use and Care of Laboratory Animals (National Research Council, Institute for Laboratory Animal Research, 2011).

### Administration of branaplam and animal tissue collection

An overview of the animal studies from which brain tissues were analyzed by immunohistochemistry and/or image analysis-based quantification is shown in Table 3.

**Table 3.**
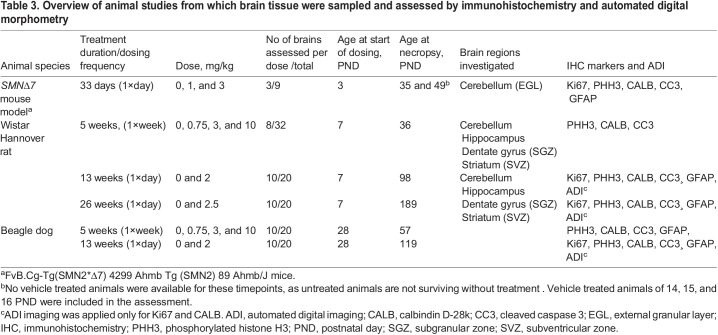
Overview of animal studies from which brain tissue were sampled and assessed by immunohistochemistry and automated digital morphometry

#### SMNΔ7 Mice

In study 1, branaplam was administered daily by oral gavage at 0.03 mg/kg/day or 3.0 mg/kg/day to neonatal male or female *SMN*Δ*7* mice, beginning on PND 3, with tissues collected at necropsy at the end of the study on PND 9. In study 2, branaplam was administered daily by oral gavage at 1.0 or 3.0 mg/kg/day to neonatal male or female *SMN*Δ*7* mice beginning on PND 3. For each study, brain tissue from nine branaplam-treated mice (three per dose group) and three vehicle-treated mice was used for immunohistochemical investigation. Tissues were collected at necropsy at the end of the study on PND 35 or PND 49. Tissues from vehicle-treated animals, treated by oral gavage in parallel in each of the two studies, were collected from study animals at necropsy on PND 9 from the first study and on PND 14, 15 or 16 in the second study.

#### Wistar Hannover rats

In study 1, branaplam was administered weekly to juvenile male or female Wistar Hannover rats by oral gavage at 0.75, 3.0 or 10 mg/kg/week, beginning on PND 7, with tissues collected at necropsy at the end of the study on PND 36. Brain tissue from 24 branaplam-treated rats (four males and four females per dose group) and from eight vehicle-treated rats (four males and four females) was used for immunnohistochemical investigation.

In study 2, branaplam was administered daily to juvenile male and female Wistar Hannover rats by oral gavage at 0.25, 0.75 or 2.0 mg/kg/day for 13 consecutive weeks, beginning on PND 7, with tissues collected at necropsy at the end of the study from high-dose animals treated with branaplam at 2.0 mg/kg/day. In study 3, branaplam was administered daily to juvenile male and female Wistar Hannover rats by oral gavage at 0.25, 0.75 or 2.5 mg/kg/day for 26 consecutive weeks, beginning on PND day 7, with tissues collected at necropsy at the end of the study from high-dose animals treated with branaplam at 2.5 mg/kg/day. Tissues from vehicle-treated animals, which were treated by oral gavage in parallel in each of the three studies, were collected from respective study animals at necropsy at the end of each respective study. For studies 2 and 3, brain tissue from ten branaplam-treated rats (five males and five females from the high-dose group) and from five vehicle-treated rats (five males and five females) was used for immunnohistochemical investigation.

#### Beagle dogs

In study 1, branaplam was administered weekly to male or female juvenile Beagle dogs by oral gavage at 0.75, 3.0 or 10 mg/kg/week, beginning on PND 28, with tissue collected at necropsy at the end of the study on PND 57. In study 2, branaplam was administered daily to male and female juvenile Beagle dogs by oral gavage at 0.25, 0.75 and 2.0 mg/kg/day, beginning on PND 28, with tissues collected at necropsy at the end of the 13-week study from high-dose animals treated with branaplam at 2.0 mg/kg/day. For immunohistochemical investigations, brain tissue was used from 30 branaplam-treated dogs (five males and five females per dose group) and ten vehicle-treated dogs (five males and five females) in the first study, and from ten branaplam-treated dogs (five males and five females from the high-dose group) and ten vehicle-treated dogs in the second study. Brain tissue from vehicle-treated dogs that were treated by oral gavage in parallel in each of the two studies was collected from respective study animals at necropsy at the end of each study.

### Tissue processing and immunohistochemistry

Sampling and processing of tissues followed best practice recommendations as outlined by the Society of Toxicologic Pathology, Nervous System Sampling Working Group (Bolon et al., 2018). Brain tissues from all animals were fixed by immersion in 10% neutral buffered formalin, trimmed, and routinely processed.

Consecutive cerebellar tissue sections of standard thickness (4 to 6 µm) from each species and tissue sections from striatum and dentate gyrus (rats and dogs) were taken from the same tissue blocks, which were used for preparation of the hematoxylin and eosin-stained tissue sections. For all studies, tissue sections from animals dosed with the highest dose and the corresponding vehicle were always included in the immunohistochemistry assessment. All sections were stained with proliferation markers (Ki67 or PHH3), Purkinje cell marker (calbindin-D28k), and apoptosis marker (cleaved caspase-3). Sections were additionally stained with a glia marker [glial fibrillary acidic protein (GFAP)] as outlined in Table 3. Cell cycle phase markers Ki67 and PHH3, Purkinje cell marker calbindin-D28k and apoptosis marker cleaved caspase-3 were applied on selected brain sections; GFAP was selected to assess possible morphological changes (Bergman glia alignment or scarring) in the cerebellum (more information on applied antibodies and specificity are shown in Table 4).

**Table 4.**
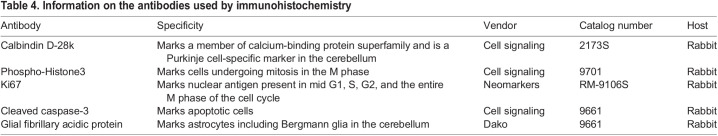
Information on the antibodies used by immunohistochemistry

Immunohistochemistry with the selected antibodies was performed using the fully automated Discovery XT^®^ instrument (Ventana Medical Systems Inc, Oro Valley, AZ, USA). For immunostaining, tissue sections acquired from paraffin blocks and mounted on glass slides were de-paraffinized and rehydrated under solvent-free conditions using EZprep™ solution for 8 min at 75°C. Tissue sections on glass slides were heated to induce epitope retrieval by successive cycles at 100°C for 4 min in a tris-ethylenediaminetetraacetic acid-based buffer optimized for the Discovery XT^®^ instrument (CC1 solution). Prior to application of respective primary antibodies, a blocking step to inhibit nonspecific antibody binding was performed by applying 1x casein solution in phosphate-buffered saline (BioFX Laboratories, Owings Mills, MD, USA; catalog number PBSC-0100-5×) for 32 min at room temperature. Endogenous avidin/biotin activity in tissues was quenched by application of Ventana A/B blocking reagents for 4 min each.

Primary antibodies were applied at the predetermined dilution to respective tissues on glass slides for 3–6 h. Secondary antibodies were applied after a short post-fixation using 0.05% glutaraldehyde. 3,3′-diaminobenzidine was used as a chromogen according to the manufacturer's recommendations (ChromoMap DAB™ or DAB Map™, Ventana Medical Systems Inc) followed by counterstaining with hematoxylin II and a bluing reagent.

### Microscopic analysis and automated digital analysis

Immunostained tissue sections were assessed by bright field light microscopy. All stained tissue sections were scanned using a Hamamatsu NanoZoomer slide scanner (NanoZoomer 2.0 HT, scanning software NDP-Scan Vers. 2.5, Hamamatsu Photonics France, Swiss Office, Solothurn, Switzerland). For image-analysis-based quantification cerebellar and striatal tissue sections from rats and dogs stained for Ki67 and calbindin D-28k were scanned using a Philips slide scanner (Philips Ultra Fast Scanner, version 1.6, Image Management System version 2.2, Philips Digital Pathology, Best, the Netherlands). A 40× objective was used for scanning, resulting in a pixel size of 0.25 µm in x and y directions. Image-analysis-based quantification was performed using Definiens Developer XD software (Developer XD 2.0, Definiens AG, Munich, Germany), and image analysis algorithms were developed for each respective immunostaining. The algorithms quantified stain-positive areas and computed unit-length labelling indices.

#### Quantification of Ki67

**Fig. 4.**
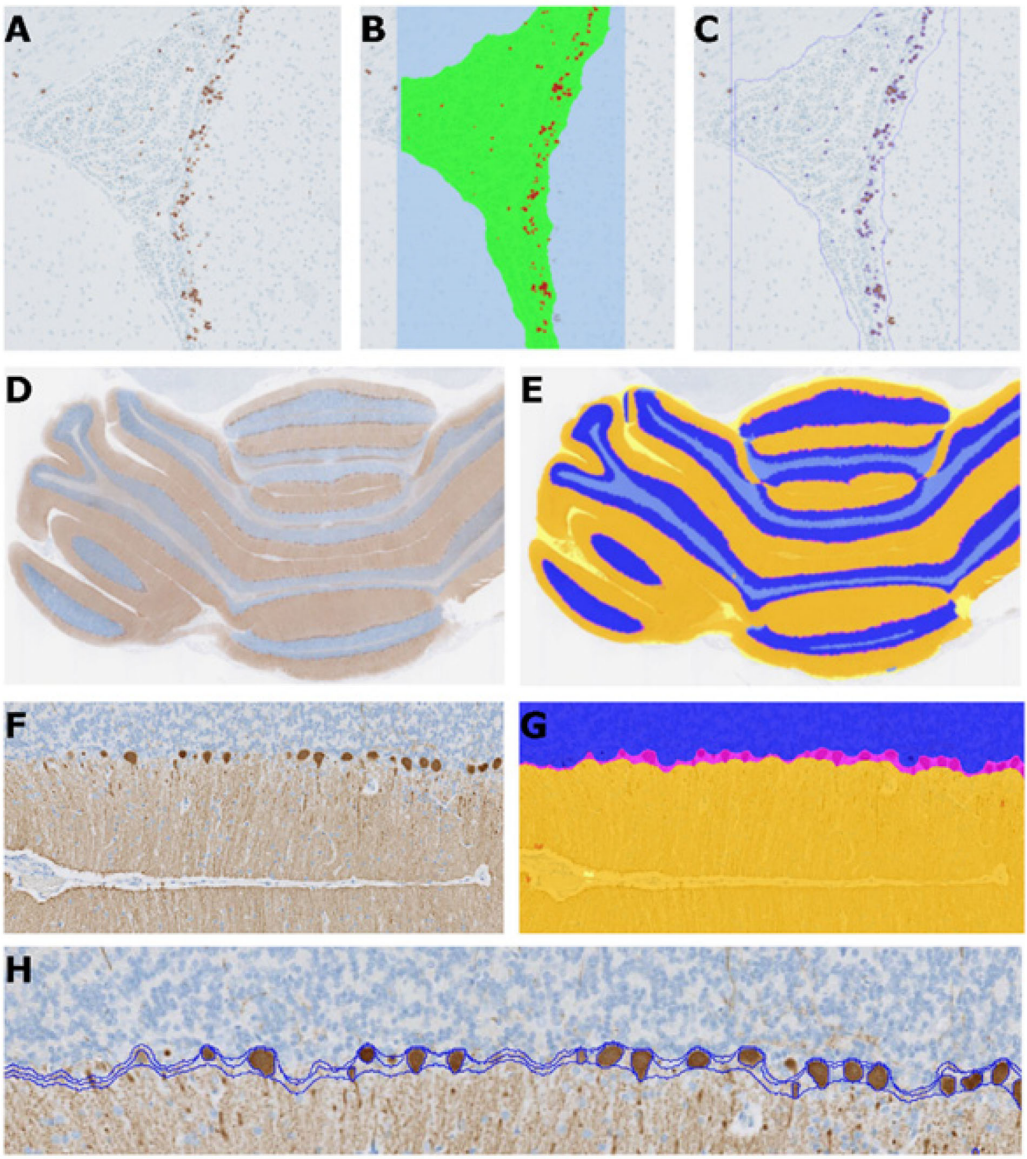
**Automated image-analysis-based quantification of cell proliferation and distribution of Purkinje cells.** Original Ki67 image (A). Image overlay showing the region of interest in green and the detected Ki67 positive areas in red (B). Outline of the detected areas (C). Original calbindin-D28k image (D). Image overlay showing the molecular layer (orange), granular cell layer (blue) and the interface area (light grey) with Purkinje cell layer (magenta) and white space (yellow) (E). Part of the original image at higher magnification, showing the molecular layer with the Purkinje cells (F). Image overlay at higher magnification (G). Outline showing the different cell layers and the interface area with the detected Purkinje cells (H).

SVZ regions in slide scans of the striatum were automatically detected as the regions of interest (see Fig. 4, panels A through C for an example of the outline of the region automatically detected in the rat). Ki67 positive staining was detected and the areas measured. The length of this region was measured and used as the denominator for the subsequent detected Ki67 positive cells. The Ki67 positive area divided by the length of the region of interested was used as a unit length labeling index (ULL index).

#### Quantification of calbindin D-28k

Slide scans of the cerebellum, the granular and molecular cell layers as well as the Purkinje cells, were detected automatically based on the positive and negative areas (see Fig. 4, panels D through G for an example of the regions automatically detected in the rat brain). The length of the molecular layer containing Purkinje cells was measured and used as the denominator for the detected Purkinje cells (see Fig. 4G for an example of the Purkinje cell nuclei detected as part of the molecular layer). The number of Purkinje cells divided by the length of the molecular layer containing Purkinje cells at the interface to the granular layer was used as the readout (cells per unit length).

### Assessment of a genotoxic potential

The potential of branaplam to induce micronucleus formation was assessed *in vitro* in human lymphocytes and *in vivo* in rats. The micronucleus test in cultured human peripheral-blood lymphocytes was performed according to ICH-S2(R1) and OECD guidelines (ICH, 2016; OECD, 2010). The *in vitro* cytokinesis-block micronucleus assay in human lymphocytes detects acentric chromosome fragments due to DNA breakage (clastogenicity) and chromosome loss (aneuploidy) resulting from chromatid/chromosome lagging in anaphase. The assay allows the assessment of cell toxicity/cell-cycle delay in combination with the cytokinesis-block method using cytochalasin B. The tests were conducted as described by Oliver et al. (2006).

Micronucleus formation *in vivo* was assessed in a dedicated study in adult male rats and as part of a 13-week repeat-dose study. In the dedicated study, adult male rats were dosed twice (24 h apart) with 1, 5 or 10 mg/kg. In a repeat-dose toxicity study, male and female juvenile rats were dosed with 0.25, 0.75 or 2.0 mg/kg/day for 13 weeks with blood sampling 24 h after the last dose and 4 weeks after discontinuation of treatment in a subset of animals. The methods used for assessing micronucleus induction in peripheral blood of rats were described by Cammerer et al. (2007).

### Pharmacokinetics

The following samples were collected for pharmacokinetic analysis of branaplam: (1) plasma and brain tissue samples from *SMN*Δ*7* mice on PND 35 or 49 (4 h post last dose) after repeated dosing at 1 and 3 mg/kg/day; (2) plasma and brain tissue samples from juvenile rats on PND 188 (24 h post last dose) after repeated dosing at 0.25, 0.75, and 2.5 mg/kg/day for 26 consecutive weeks starting dosing at PND 7; and (3) plasma, brain tissue, and CSF samples from juvenile dogs on PND 119 (24 h post last dose) after repeated dosing to 0.25, 0.75, and 2 mg/kg/day for 13 consecutive weeks starting dosing at PND 28.

A qualified LC-MS/MS method with a dynamic range of 1–1000 ng/ml was employed for the analysis of branaplam in the plasma and brain tissue samples collected from *SMN*Δ*7* mice. Protein precipitation was used for processing the plasma samples and brain tissue homogenate prior to LC-MS/MS analysis on a Sciex QTRAP5500 system (Sciex, Toronto, Canada) coupled with Agilent 1200 HPLC pumps. Chromatographic separation was carried out on a MAC-MOD C_18_ (30×2.1 mm, 3 µm particle size) column with a flow rate of 0.7 ml/min and column temperature set at 50°C. The mobile phase A was water containing 0.1% formic acid and mobile phase B was acetonitrile containing 0.1% formic acid. Gradient elution was as follows: 0 min, 5% B; 0.3 min, 95% B; 0.3 to 1.8 min 95% B, 2.5 min, 5%B and 3.0 min, stop. Electrospray in positive ionization mode with multiple-reaction monitoring (MRM) was employed for the MS/MS detection at the following mass transitions: (i) branaplam: *m/z* 394.2→140.2; (ii) internal standard gluburide: *m/z* 494.4→369.0.

Fully validated LC-MS/MS methods were implemented in the analysis of branaplam in the plasma samples (0.1 to 100 ng/ml for rat plasma and 0.2 to 200 ng/ml for dog plasma) while qualified LC-MS/MS methods were employed for the analysis of the compound in the CSF (0.2 to 200 ng/ml) and brain tissue (0.5 to 500 ng/***g*** for rats and 1 to 1000 ng/***g*** for dogs) samples collected from the juvenile rats and juvenile dogs. In these measurements, plasma, CSF (diluted to 1:1 with plasma), and homogenized brain samples (diluted to 1:4 with PBS buffer) were processed using protein precipitation method with acetonitrile:methanol (90:10, v:v). Upon vortex-mixing and centrifugation, the resulting supernatants was dried under nitrogen stream. The reconstituted sample extract were submitted to LC-MS/MS analysis on a Sceix QTRAP 6500 system (Sciex, Toronto, Canada) coupled with Shimadzu HPLC pumps. Chromatographic separation was carried out on a MAC-MOD C_18_ (3 μm, 4.6×50 mm) column with a flow rate of 0.8 ml/min and a column temperature set at 40°C. The mobile phase was water:acetonitrile (95:5, v:v) containing 0.5% acetic acid and 5 mM ammonium acetate and mobile B was acetonitrile containing 0.5% acetic acid. Gradient elution was as follows: 0 to 2 min 26% B; 2.0 to 2.01 min, 26% to 95% B; 2.01 to 2.5 min 95% B, 0.50 to 2.51 min, 95 to 26% B; 2.51 to 3.2 min 26% B. Mass transitions of (i) *m/z* 394.3→140.3 for branaplam and (ii) *m/z* 400.3→140.3 for the internal standard [(M+6) branaplam] were employed in the MS detection under positive electrospray ionization with multiple-reaction monitoring.

The LC-MS/MS data from the above analysis were processed with Analyst software (versions 1.6 and 1.6.2, Applied Biosystems, Foster City, CA, USA). The analyte concentration data was calculated using Analyst software or Watson LIMS (version 7.4.1 and 7.4.2, Thermo Fisher Scientific, Philadelphia, PN, USA).

## Supplementary Material

Supplementary information

## References

[BIO058551C1] Altman, J. and Bayer, S. A. (1990). Mosaic organization of the hippocampal neuroepithelium and the multiple germinal sources of dentate granule cells. *J. Comp. Neurol.* 301, 325-342. 10.1002/cne.903010302 2262594

[BIO058551C2] Bolon, B., Krinke, G., Butt, M. T., Rao, D. B., Pardo, I. D., Jortner, B. S., Garman, R. H., Jensen, K., Andrews-Jones, L., Morrison, J. P. et al. (2018). STP position paper: recommended best practices for sampling, processing, and analysis of the peripheral nervous system (nerves and somatic and autonomic ganglia) during nonclinical toxicity studies. *Toxicol. Pathol.* 46, 372-402. 10.1177/0192623318772484 29787347

[BIO058551C3] Burghes, A. H. M. and Beattie, C. E. (2009). Spinal muscular atrophy: why do low levels of survival motor neuron protein make motor neurons sick? *Nat. Rev. Neurosci.* 10, 597-609. 10.1038/nrn2670 19584893 PMC2853768

[BIO058551C4] Cammerer, Z., Elhajouji, A., Kirsch-Volders, M. and Suter, W. (2007). Comparison of the peripheral blood micronucleus test using flow cytometry in rat and mouse exposed to aneugens after single-dose applications. *Mutagenesis* 22, 129-134. 10.1093/mutage/gel066 17284774

[BIO058551C5] Charnas, L., Voltz, E., Pfister, C., Peters, T., Hartmann, A., Berghs-Clairmont, C., Praestgaard, J., de Raspide, M., Deconinck, N., Born, A. et al. (2017). Safety and efficacy findings in the first-in-human trial (FIH) of the oral splice modulator branaplam in type 1 spinal muscular atrophy (SMA): interim results. *Neuromuscul. Disord.* 27, S207-S208. 10.1016/j.nmd.2017.06.411

[BIO058551C6] Cheung, A. K., Hurley, B., Kerrigan, R., Shu, L., Chin, D. N., Shen, Y., O'Brien, G., Sung, M. J., Hou, Y., Axford, J. et al. (2018). Discovery of small molecule splicing modulators of survival motor neuron-2 (SMN2) for the treatment of spinal muscular atrophy (SMA). *J. Med. Chem.* 61, 11021-11036. 10.1021/acs.jmedchem.8b01291 30407821

[BIO058551C7] Day, J., Annoussamy, M., Baranello, G., Boespflug-Tanguy, O., Borell, S., Goemans, N., Kirschner, J., Masson, R., Pera, M., Servais, L. et al. (2020). Sunfish part 1: 24-month safety and exploratory outcomes of risdiplam (RG7916) TREATMENT IN PATIENTS WITH TYPE 2 OR 3 SPINAL MUSCULAR ATROPHY (SMA). In 25th International Annual Congress of the World Muscle Society: PTC Therapeutics, Inc.

[BIO058551C8] Dunant, N. (2020). Roche's Risdiplam meets primary endpoint in pivotal FIREFISH trial in infants with type 1 spinal muscular atrophy, (ed. The Roche Group https://www.roche.com/media/releases/med-cor-2020-01-23.htm).

[BIO058551C9] FDA News Release (2020). FDA Approves Oral Treatment for Spinal Muscular Atrophy. https://www.fda.gov/news-events/press-announcements/fda-approves-oral-treatment-spinal-muscular-atrophy.

[BIO058551C10] Fuentealba, L. C., Obernier, K. and Alvarez-Buylla, A. (2012). Adult neural stem cells bridge their niche. *Cell Stem Cell* 10, 698-708. 10.1016/j.stem.2012.05.012 22704510 PMC3726005

[BIO058551C11] Gadsdon, D. R. and Emery, J. L. (1976). Some quantitative morphological aspects of post-natal human cerebellar growth. *J. Neurol. Sci.* 29, 137-148. 10.1016/0022-510X(76)90166-0 978206

[BIO058551C12] Garman, R. H., Li, A. A., Kaufmann, W., Auer, R. N. and Bolon, B. (2015). Recommended Methods for Brain Processing and Quantitative Analysis in Rodent Developmental Neurotoxicity Studies. *Toxicol. Pathol.* 44, 14-42. 10.1177/0192623315596858 26296631

[BIO058551C13] Heinsen, H. (1977). Quantitative anatomical studies on the postnatal development of the cerebellum of the albino rat. *Anat. Embryol.* 151, 201-218. 10.1007/BF00297481 920968

[BIO058551C14] Heng, C., Lefebvre, O., Klein, A., Edwards, M. M., Simon-Assmann, P., Orend, G. and Bagnard, D. (2011). Functional role of laminin α1 chain during cerebellum development. *Cell adhesion & migration* 5, 480-489. 10.4161/cam.5.6.19191 22274713 PMC3277781

[BIO058551C15] Jevtic, S., Carr, D. and Dobrzycka-Ambrozevicz, A. (2019). Branaplam in Type 1 spinal muscular atrophy: second part of a phase I/II Study. In Communication presented at 23rd SMA researcher meeting, Cure SMA. Anaheim, CA, USA.

[BIO058551C16] Kolb, S. J. and Kissel, J. T. (2015). Spinal muscular atrophy. *Neurol. Clin.* 33, 831-846. 10.1016/j.ncl.2015.07.004 26515624 PMC4628728

[BIO058551C17] Le, T. T., Pham, L. T., Butchbach, M. E. R., Zhang, H. L., Monani, U. R., Coovert, D. D., Gavrilina, T. O., Xing, L., Bassell, G. J. and Burghes, A. H. M. (2005). SMNΔ7, the major product of the centromeric survival motor neuron (SMN2) gene, extends survival in mice with spinal muscular atrophy and associates with full-length SMN. *Hum. Mol. Genet.* 14, 845-857. 10.1093/hmg/ddi078 15703193

[BIO058551C18] Li, A. A., Sheets, L. P., Raffaele, K., Moser, V., Hofstra, A., Hoberman, A., Makris, S. L., Garman, R., Bolon, B., Kaufmann, W. et al. (2017). Recommendations for harmonization of data collection and analysis of developmental neurotoxicity endpoints in regulatory guideline studies: proceedings of workshops presented at society of toxicology and joint teratology society and neurobehavioral teratology society meetings. *Neurotoxicol. Teratol.* 63, 24-45. 10.1016/j.ntt.2017.07.001 28757310 PMC6634984

[BIO058551C19] Lunn, M. R. and Wang, C. H. (2008). Spinal muscular atrophy. *The Lancet* 371, 2120-2133. 10.1016/S0140-6736(08)60921-6 18572081

[BIO058551C20] Marzban, H., Del Bigio, M. R., Alizadeh, J., Ghavami, S., Zachariah, R. M. and Rastegar, M. (2015). Cellular commitment in the developing cerebellum. *Front. Cell. Neurosci.* 8, 450-450. 10.3389/fncel.2014.00450 25628535 PMC4290586

[BIO058551C21] Ming, G.-l. and Song, H. (2011). Adult neurogenesis in the mammalian brain: significant answers and significant questions. *Neuron* 70, 687-702. 10.1016/j.neuron.2011.05.001 21609825 PMC3106107

[BIO058551C22] Ohmori, H., Ogura, H., Yasuda, M., Nakamura, S., Hatta, T., Kawano, K., Michikawa, T., Yamashita, K. and Mikoshiba, K. (1999). Developmental neurotoxicity of phenytoin on granule cells and purkinje cells in mouse cerebellum. *J. Neurochem.* 72, 1497-1506. 10.1046/j.1471-4159.1999.721497.x 10098854

[BIO058551C23] Oliver, J., Meunier, J.-R., Awogi, T., Elhajouji, A., Ouldelhkim, M.-C., Bichet, N., Thybaud, V., Lorenzon, G., Marzin, D. and Lorge, E. (2006). SFTG international collaborative study on in vitro micronucleus test: V. Using L5178Y cells. *Mutat. Res.* 607, 125-152. 10.1016/j.mrgentox.2006.04.004 16797225

[BIO058551C24] Organization for Economic Co-operation and Development (2007). *Test No. 426: Developmental Neurotoxicity Study, OECD Guidelines for the Testing of Chemicals Paris*. France: OECD Publishing.

[BIO058551C25] Organization for Economic Co-operation and Development (2011). *Test No. 443: Extended One-Generation Reproductive Toxicity Study*. Paris, France: OECD Publishing.

[BIO058551C26] Organization for Economic Co-operation and Development (2015). *Test No. 422: Combined Repeated Dose Toxicity Study with the Reproduction/Developmental Toxicity Screening Test*. Paris, France: OECD Publishing.

[BIO058551C27] Palacino, J., Swalley, S. E., Song, C., Cheung, A. K., Shu, L., Zhang, X., Van Hoosear, M., Shin, Y., Chin, D. N., Keller, C. G. et al. (2015). SMN2 splice modulators enhance U1–pre-mRNA association and rescue SMA mice. *Nat. Chem. Biol.* 11, 511-517. 10.1038/nchembio.1837 26030728

[BIO058551C28] Phemister, R. D. and Young, S. (1968). The postnatal development of the canine cerebellar cortex. *J. Comp. Neurol.* 134, 243-253. 10.1002/cne.901340209 5712418

[BIO058551C29] Pisu, M. B., Roda, E., Guioli, S., Avella, D., Bottone, M. G. and Bernocchi, G. (2005). Proliferation and migration of granule cells in the developing rat cerebellum: Cisplatin effects. *Anat. Rec. A Discov. Mol. Cell. Evol. Biol.* 287A, 1226-1235. 10.1002/ar.a.20249 16247801

[BIO058551C30] Ramdas, S. and Servais, L. (2020). New treatments in spinal muscular atrophy: an overview of currently available data. *Expert Opin Pharmacother.* 21, 307-315. 10.1080/14656566.2019.1704732 31973611

[BIO058551C31] Ratni, H., Ebeling, M., Baird, J., Bendels, S., Bylund, J., Chen, K. S., Denk, N., Feng, Z., Green, L., Guerard, M. et al. (2018). Discovery of risdiplam, a selective survival of motor neuron-2 (SMN2) gene splicing modifier for the treatment of spinal muscular atrophy (SMA). *J. Med. Chem.* 61, 6501-6517. 10.1021/acs.jmedchem.8b00741 30044619

[BIO058551C32] Risdiplam Prescribing Information. (2020). Available at https://www.accessdata.fda.gov/drugsatfda_docs/label/2020/213535s000lbl.pdf. Accessed October 2021.

[BIO058551C33] Shigemoto-Mogami, Y., Hoshikawa, K., Goldman, J. E., Sekino, Y. and Sato, K. (2014). Microglia enhance neurogenesis and oligodendrogenesis in the early postnatal subventricular zone. *J. Neurosci.* 34, 2231-2243. 10.1523/JNEUROSCI.1619-13.2014 24501362 PMC3913870

[BIO058551C34] Sorrells, S. F., Paredes, M. F., Cebrian-Silla, A., Sandoval, K., Qi, D., Kelley, K. W., James, D., Mayer, S., Chang, J., Auguste, K. I. et al. (2018). Human hippocampal neurogenesis drops sharply in children to undetectable levels in adults. *Nature* 555, 377-381. 10.1038/nature25975 29513649 PMC6179355

[BIO058551C35] Spinraza Prescribing Information. (2021). Available at https://www.accessdata.fda.gov/drugsatfda_docs/label/2016/209531lbl.pdf. Accessed October 2021.

[BIO058551C36] Sugarman, E. A., Nagan, N., Zhu, H., Akmaev, V. R., Zhou, Z., Rohlfs, E. M., Flynn, K., Hendrickson, B. C., Scholl, T., Sirko-Osadsa, D. A. et al. (2012). Pan-ethnic carrier screening and prenatal diagnosis for spinal muscular atrophy: clinical laboratory analysis of >72400 specimens. *Eur. J. Hum. Genet.* 20, 27-32. 10.1038/ejhg.2011.134 21811307 PMC3234503

[BIO058551C37] Swoboda, K. J., Prior, T. W., Scott, C. B., McNaught, T. P., Wride, M. C., Reyna, S. P. and Bromberg, M. B. (2005). Natural history of denervation in SMA: relation to age, SMN2 copy number, and function. *Ann. Neurol.* 57, 704-712. 15852397 10.1002/ana.20473 PMC4334582

[BIO058551C38] Tizzano, E. F. and Finkel, R. S. (2017). Spinal muscular atrophy: a changing phenotype beyond the clinical trials. *Neuromuscul. Disord.* 27, 883-889. 10.1016/j.nmd.2017.05.011 28757001

[BIO058551C39] United States Environmental Protection Agency (1998). Neurotoxicity screening battery. In *Health Effects Test Guidelines*, pp. OPPTS 870.6200. Washington, DC, USA: USEPA.

[BIO058551C40] Watson, R. E., DeSesso, J. M., Hurtt, M. E. and Cappon, G. D. (2006). Postnatal growth and morphological development of the brain: a species comparison. *Birth Defects Res. B Dev. Reprod. Toxicol.* 77, 471-484. 10.1002/bdrb.20090 17066419

[BIO058551C41] Zolgensma Prescribing Information. (2021). Available at https://www.fda.gov/media/126109/download. Accessed October 2021.

