# Supplementary material for: Orally administered branaplam does not impact neurogenesis in juvenile mice, rats, and dogs
Source: Biol Open. 2021 Oct 28;10(10):bio058551. doi: 10.1242/bio.058551 (PMC8565466; doi:10.1242/bio.058551)
Supplement: Supplementary information [file biolopen-10-058551-s1.pdf]

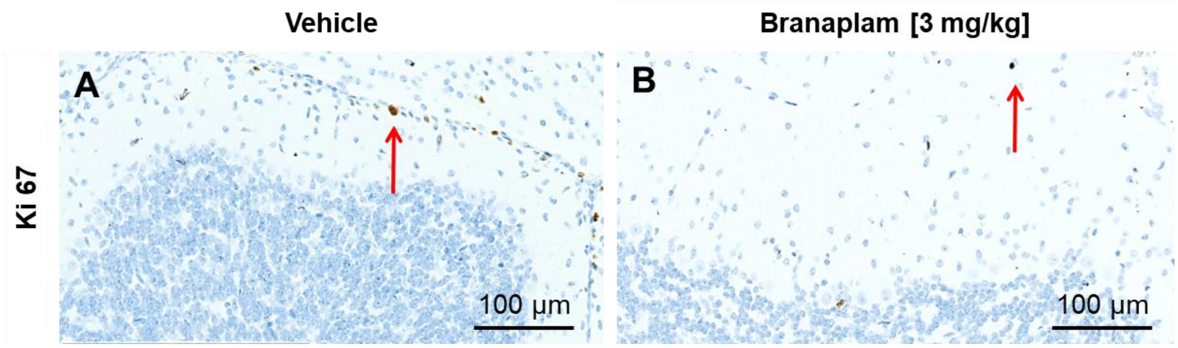

Fig. S1. Immunohistochemistry in the cerebellum of mice with SMA. Ki67 staining at the level of the former extragranaular layer (EGL, red arrows) in vehicle on post natal day (PND) 14 (A) and branaplam (B) treated mice on PND 35.

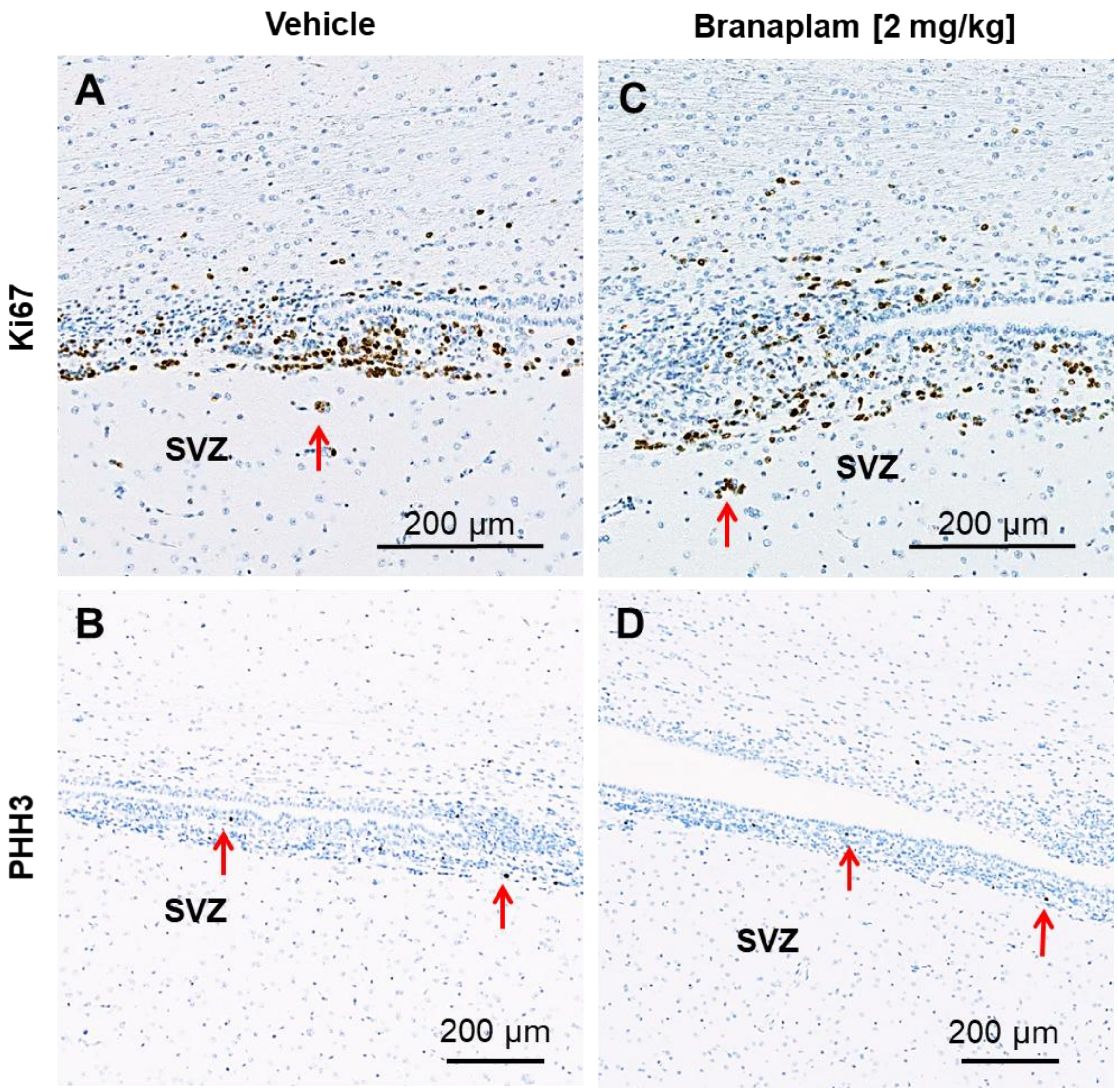

Fig. S2. Immunohistochemistry in the striatum of dogs at the level of the subventricular zone (SVZ). Ki67 and PHH3 staining (red arrows) in vehicle (A and B) and in branaplam (C and D) treated dogs on PND 119.

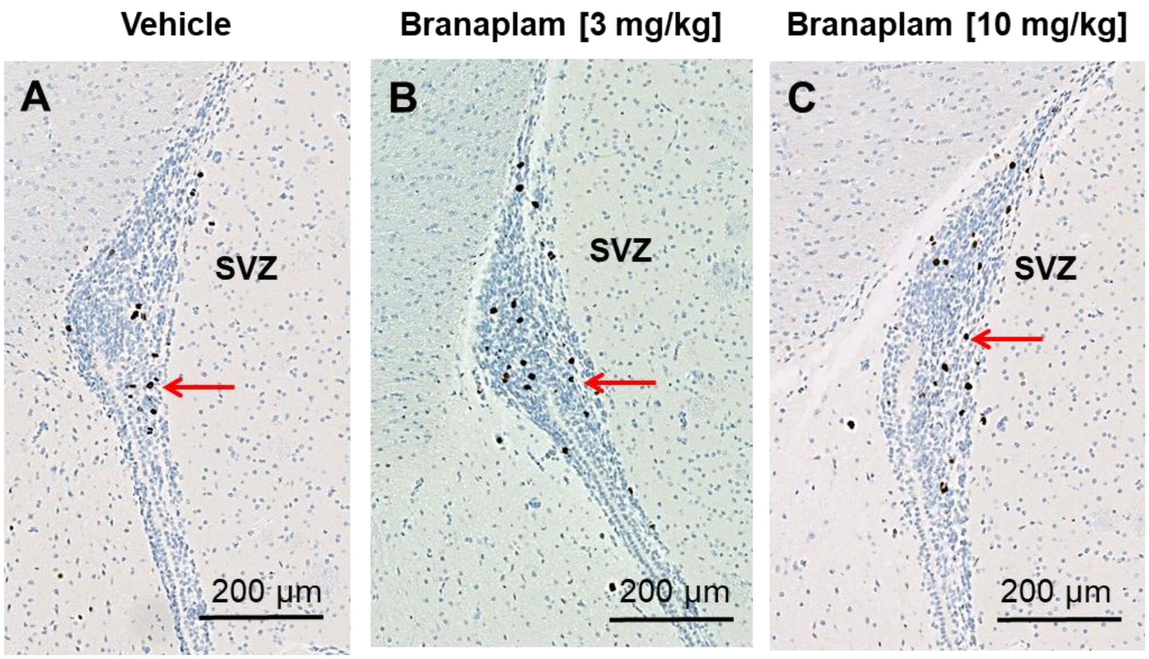

Fig. S3A. Immunohistochemistry in the striatum of rats at the level of the subventricular zone (SVZ). Panel A, B, and C show PHH3 staining comparing proliferating cells (M-phase, red arrows) in rats at PND 36.

Biology Open • Supplementary information

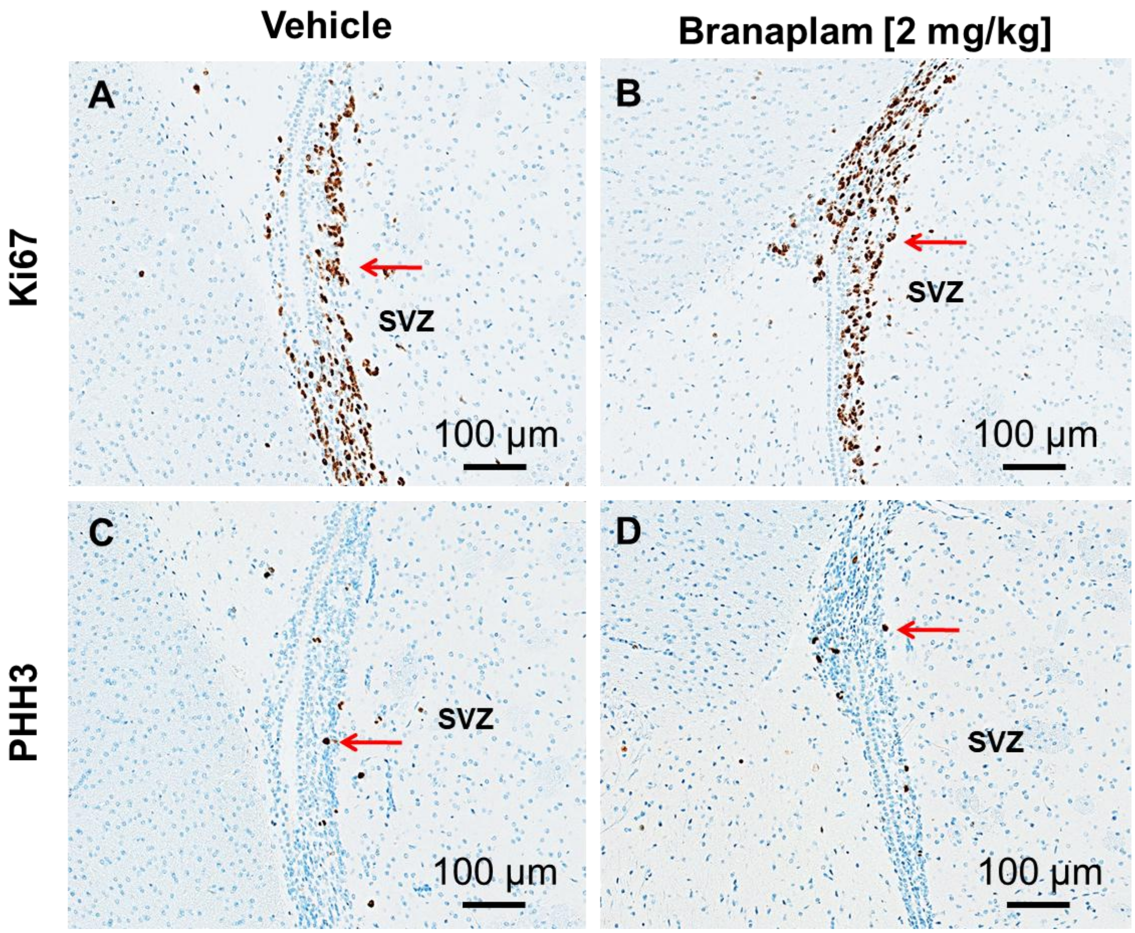

Fig. S3B. Immunohistochemistry in the striatum of rats at the level of the subventricular zone (SVZ). Ki67 and PHH3 staining (red arrows) in vehicle (A and C) and in branaplam (B and D) treated rats on PND 98.

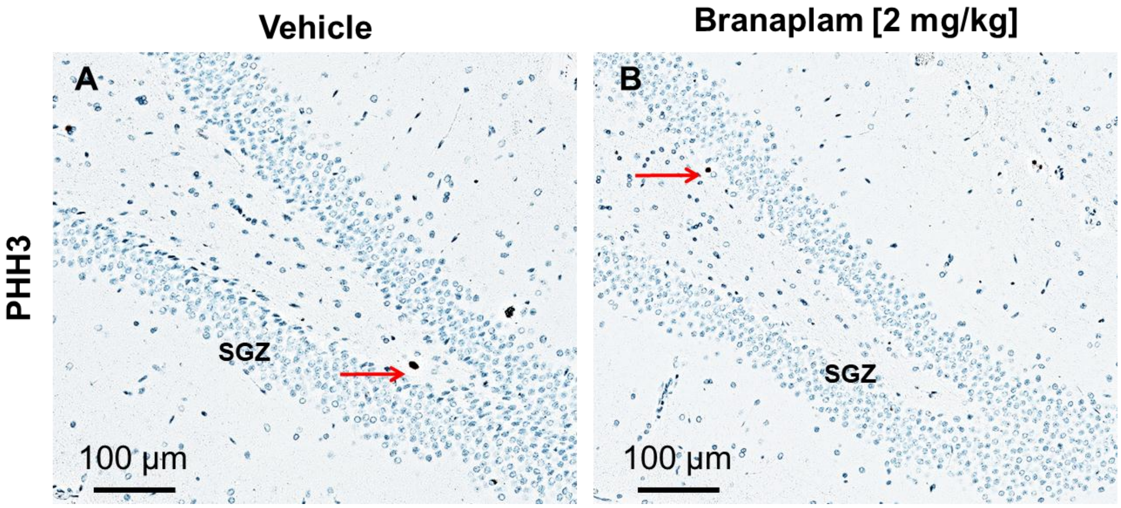

Fig. S3C. Immunohistochemistry in the Subgranular Zone (SGZ) of rats. PHH3 staining (red arrows) in vehicle (A) and branaplam (B) treated rats on PND 98.

Biology Open • Supplementary information

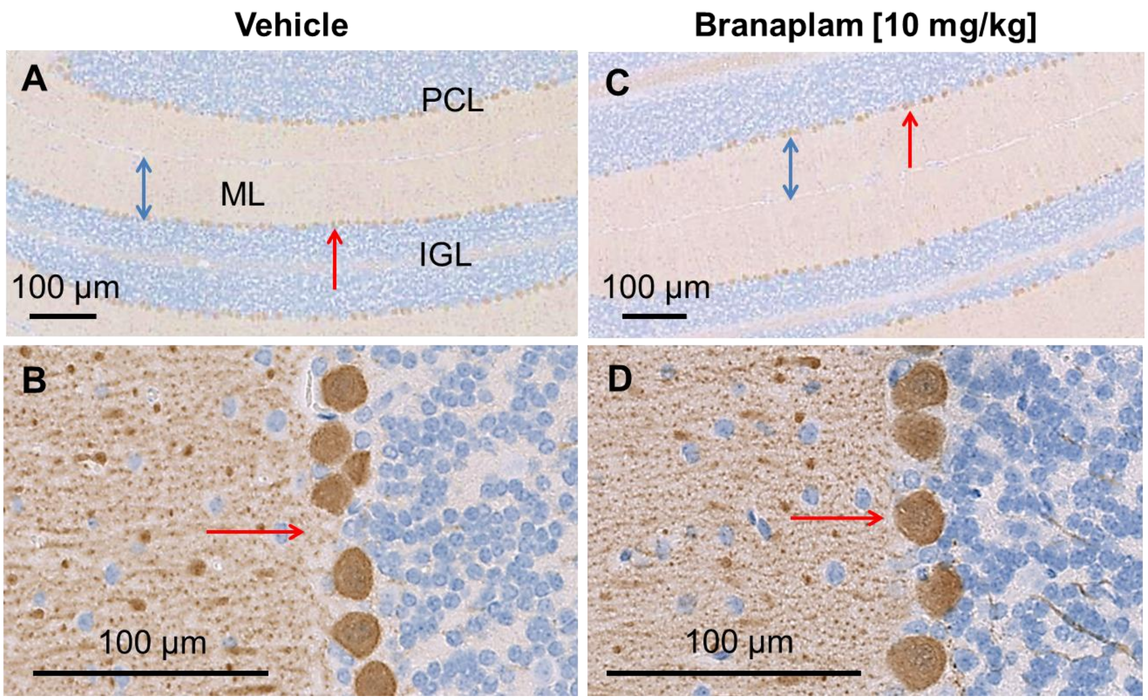

Fig. S4A. Immunohistochemistry in the cerebellum of rats. Calbindin staining in vehicle (A, B) and branplam (C, D) treated rats on PND 36. Red arrows point to normal aligned Purkinje cells. Two-headed blue arrows designate molecular layer width. PCL, Purkinje cell layer; ML, molecular layer.

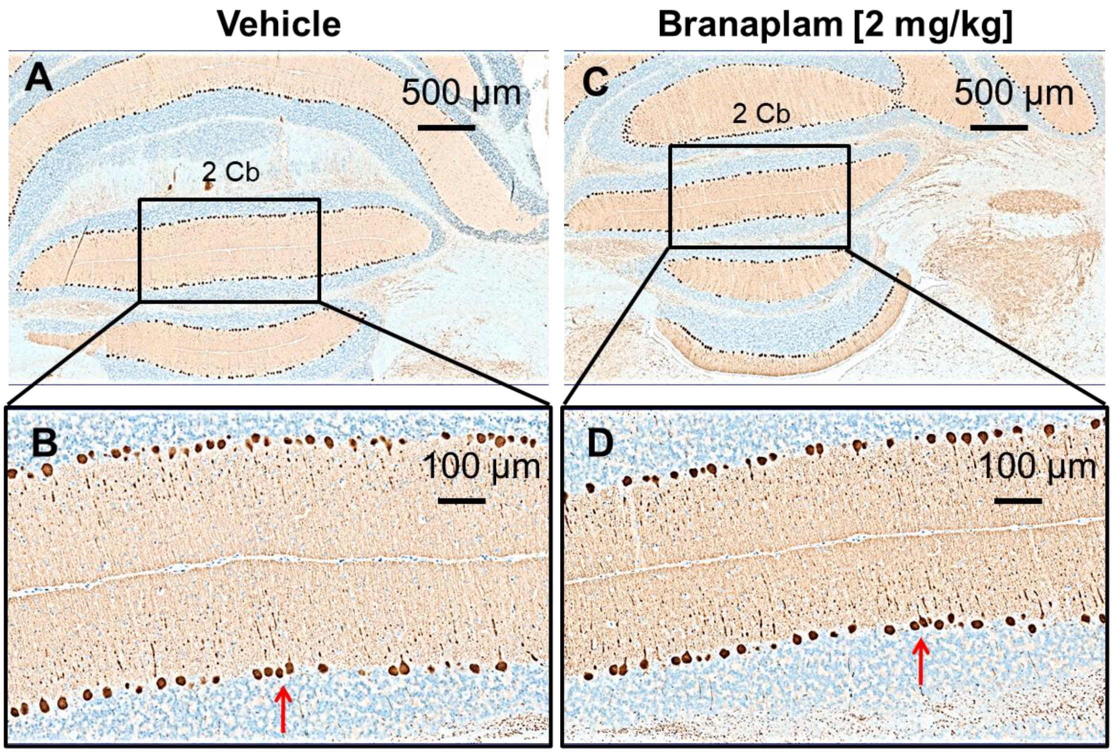

Fig. S4B. Immunohistochemistry in the cerebellum of rats. Calbindin staining in vehicle (A, B) and branplam (C, D) treated rats on PND 98. Rectangles mark the lobule 2 of the cerebellar vermis (2Cb). Red arrows point to normal aligned Purkinje cells in higher magnification pictures from the rectangles.

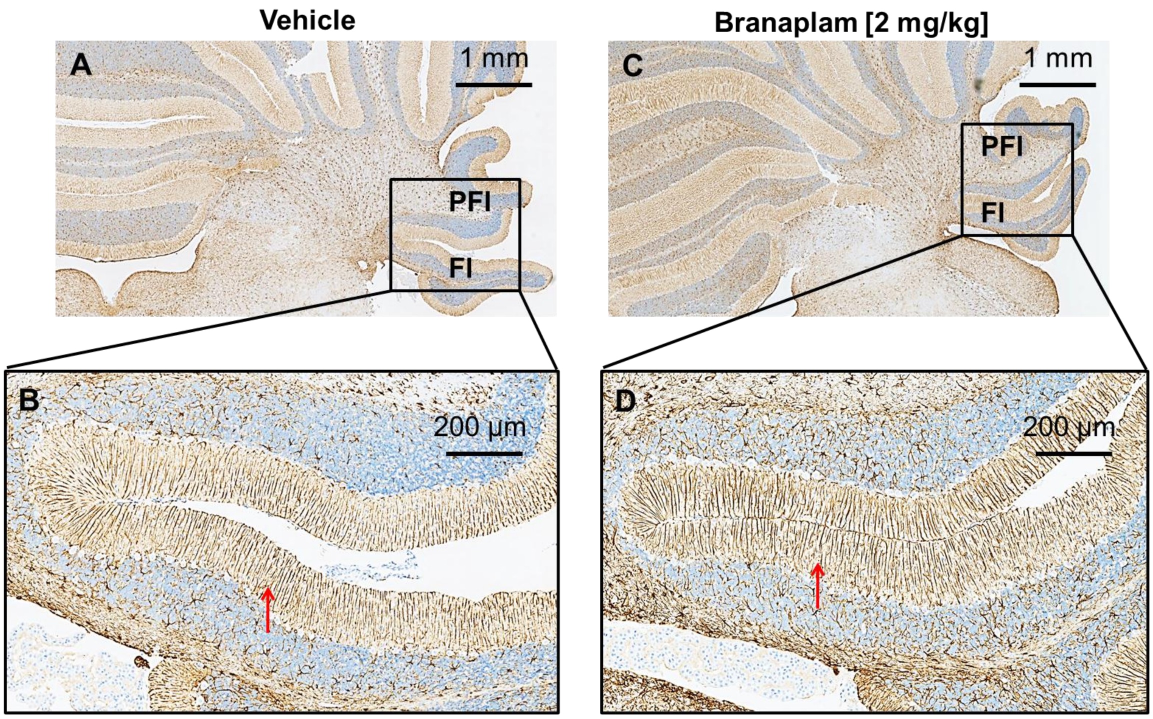

Fig. S4C. Immunohistochemistry in the cerebellum of rats. GFAP staining in vehicle (A and B) and in branaplam (C and D) treated rats on PND 98. Rectangles mark the Paraflocculus (PFl) and Flocculus (Fl) regions where late postnatal neurogenesis occurs. Arrows point to normal parallel Bergmann glia fibers in higher magnification pictures from the rectangles.

Biology Open • Supplementary information

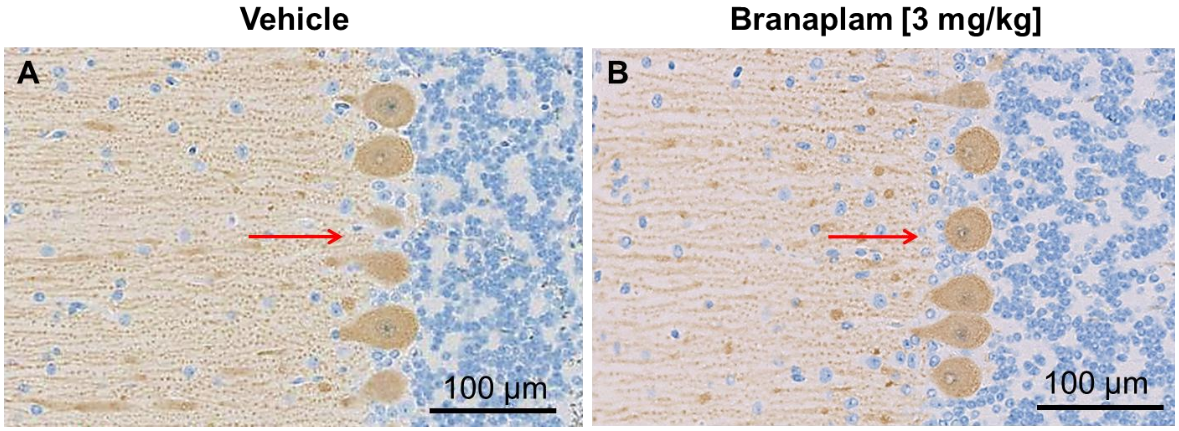

Fig. S5A. Immunohistochemistry in the cerebellum of dogs. Calbindin staining in the vehicle (A) and branaplam (B) treated dogs on PND 57. Red arrows point to normal aligned Purkinje cells.

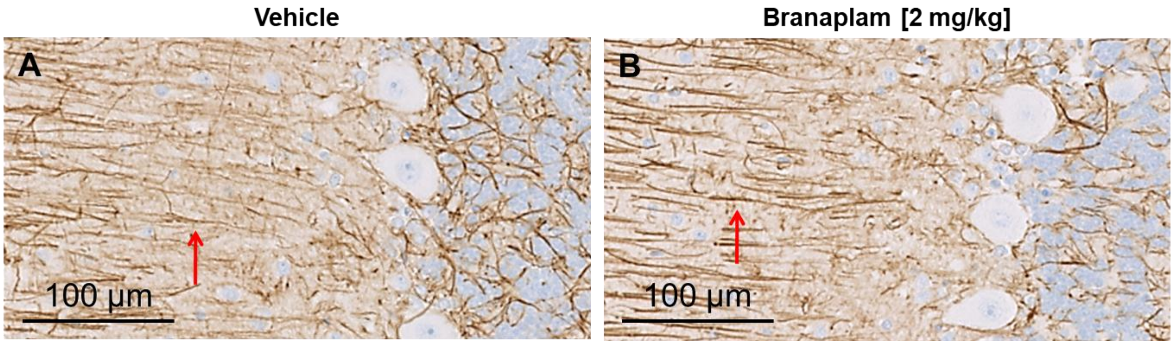

Fig. S5B. Immunohistochemistry in the cerebellum of dogs. GFAP staining in vehicle (A) and branaplam (B) treated dog on PND 119, showing normal arrangement of Bergman glial fibers (red arrows).

Biology Open • Supplementary information
